# Association between use of oral-antidiabetic drugs and the risk of aortic aneurysm: a nested case–control analysis

**DOI:** 10.1186/s12933-016-0447-9

**Published:** 2016-09-01

**Authors:** Chien-Yi Hsu, Yu-Wen Su, Yung-Tai Chen, Shih-Hung Tsai, Chun-Chin Chang, Szu-Yuan Li, Po-Hsun Huang, Jaw-Wen Chen, Shing-Jong Lin

**Affiliations:** 1 Division of Cardiology, Department of Medicine, Taipei Veterans General Hospital, Taipei, Taiwan; 2 Division of Endocrinology and Metabolism, Department of Medicine, Taipei Veterans General Hospital, Taipei, Taiwan; 3 Department of Medical Research, Taipei Veterans General Hospital, 112, No. 201, Sec. 2, Shih-Pai Road, Taipei, Taiwan; 4 Division of Nephrology, Department of Medicine, Taipei Veterans General Hospital, Taipei, Taiwan; 5 Institute of Clinical Medicine, National Yang-Ming University, Taipei, Taiwan; 6 Institute of Pharmacology, National Yang-Ming University, Taipei, Taiwan; 7 Cardiovascular Research Center, National Yang-Ming University, Taipei, Taiwan; 8 Department of Emergency Medicine, Tri-Service General Hospital, National Defense Medical Center, No.325, Sec.2, Cheng-Kung Road, Taipei, Taiwan; 9 Department of Medicine, Taipei City Hospital, Heping Fuyou Branch, Taipei, Taiwan; 10 Taipei Veterans General Hospital, Taoyuan Branch, Taoyuan, Taiwan; 11 Department of Internal Medicine, School of Medicine, College of Medicine, Taipei Medical University, Taipei, Taiwan; 12 Division of Cardiology and Cardiovascular Research Center, Department of Internal Medicine, Taipei Medical University Hospital, Taipei, Taiwan

**Keywords:** Diabetes mellitus, Oral antidiabetic drugs, Aortic aneurysm

## Abstract

**Background:**

Pleiotropic effects on cardiovascular protection have been suggested in several oral antidiabetic drugs (OAD). The impacts of OADs on aortic aneurysm (AA) growth have been found in animal studies, but the evidence of their beneficial effects for AA protection in human are lacking. We investigated the relationship between OAD therapy and the risk of developing AA.

**Methods:**

We conducted a nested case–control analysis using the database extracted from Taiwan’s National Health Insurance Research Database. The database consists of 1.2 million diabetic patients representing the majority of the type 2 diabetes population in Taiwan from 2000 to 2013. Cases were identified as those with either inpatient or outpatient diagnosis code of AA. One control was selected for each case matching on duration of follow-up, age, sex, urbanization, monthly income, severity of diabetes, and risk factor for AA. We identified variable classes of OADs, including metformin, sulfonylureas, thiazolidinedione (TZD), alpha-glucosidase inhibitors, meglitinide, dipeptidyl peptidase-4 (DPP-4) inhibitors prior to the development of AA.

**Results:**

A total of 4468 cases diagnosed with AA and 4468 matched controls were identified. Metformin use, sulfonylurea use, and TZD were associated with lower risk of developing AA, odds ratio [OR] 0.72 (95 % confidence interval [CI] 0.64–0.80), 0.82 (95 % CI 0.74–0.92), and 0.82 (95 % CI 0.69–0.98), respectively. The effects of metformin and sulfonylurea on AA were dose responsive. Neither alpha-glucosidase inhibitors (OR 0.95; 95 % CI 0.81–1.11) nor DPP-4 inhibitors (OR 0.85; 95 % CI 0.68–1.07) was significantly associated with AA events.

**Conclusions:**

Metformin, sulfonylurea, and TZD treated patients were associated with lower risks of AA development, but not DPP-4 inhibitors or alpha-glucosidase inhibitor. The protective effects of hypoglycemic agents are further confirmed by the dose responsive relations in metformin and sulfonylurea groups.

## Background

Aortic aneurysm (AA) is a potentially life-threatening disease while progressing to aneurysm rupture. There are several well-established risk factors for AA development, including older age, male gender, hypertension, Caucasian race, genetic factors, waist circumference, and other atherosclerotic diseases [1–4]. The hallmark of AA development is aortic vessel wall protein destruction, and subsequent transmural wall expansion. The process results from inflammation, oxidative stress, apoptosis, and proteolysis within extracellular matrix [5, 6]. A recent prospective study reported higher levels of coronary vascular disease biomarkers, including high-sensitivity C-reactive protein and heart-type fatty acid-binding protein, was found in patients with small or medium size AAs [7]. Based on these results, the authors suggested that AAs might be regarded as subclinical atherosclerosis.

Pleiotropic effects on cardiovascular protection have been suggested in several oral antidiabetic drugs (OAD). Metformin, one of the oldest drugs for diabetes treatment, was shown to inhibit aortic smooth muscle cell proliferation, and matrix metalloprotein (MMP)-2 expression in experimental studies [8]. The thiazolidinediones (TZD) modulate peroxisome proliferator-activated receptor-γ, a nuclear hormone receptor family transcription factor, which affects MMP-9 activity, and release of cytokines [9]. Another class of OAD, dipeptidyl peptidase-4 (DPP-4) inhibitors, also decreases production of reactive oxygen species (ROS) in cardiac mitochondria [10]. Whether the OAD-associated decrease in inflammation, MMP activities, and ROS production protects aortic vessel from aneurysm formation remains uncertain. Previous animal studies have tested this hypothesis by treating apolipoprotein-E (ApoE) deficient mice with metformin [11], pioglitazone [12], rosiglitazone [9], and DPP-4 inhibitors alogliptin [13] and sitagliptin [14]. Development and enlargement of AA decreased in the OAD-treated mice. The results were compatible with the hypothesis.

AA is a disease of relative low incidence rate in general population. Therefore, it is difficult to conduct a randomize control study with sufficient power to investigate the correlations between OAD use and development of AA. Thus, we designed a nested-case control analysis in a real world database aim to evaluate the impact of OADs on AA occurrence.

## Methods

### Data source

The Taiwan National Health Insurance (NHI) program is a social insurance program organized by the government. The program was launched in 1995, providing comprehensive medical care, including outpatient care, emergency department care, hospital care, dental services, medical examinations, laboratory tests, medication prescriptions, and interventional procedures. It is compulsory for all citizens from birth, and therefore covers nearly all (98.4 %) of Taiwan’s population. Except for healthcare services, the NHI Administration was also in charge of the collected database of all available records from individuals involved in the insurance, composing the National Health Insurance Research Database (NHIRD) [15]. This database was released for research purposes after encryption and deidentification with removing patient’s personal information to protect individual privacy. Numerous high quality scientific research papers have been published using data from NHIRD [16, 17].

For this study, we used the Longitudinal Cohort of Diabetes Patients data set. It is validated by the Taiwan National Health Research Institutes for research purposes [18]. This database represents most of the population of diabetic mellitus patients in Taiwan, with a sample of total 1,200,000 patients diagnosed with diabetes since 1999 [19]. It consists of deidentified secondary data for patient’s privacy protection. This study was approved by the institutional review board of Taipei City Hospital (TCHIRB-10404107-W), and written informed consent of patients was waived. The diseases were defined by the International Classification of Disease, Ninth Revision, Clinical Modification (ICD-9-CM) diagnosis codes, 2001 edition.

### Participants and control

In the nested case–control analysis, we aimed to identify the association between OAD use and developing of AA. We extracted all diabetes patients with age ≥20 years between January 2000 and December 2013. Cases were identified as those with either inpatient or outpatient diagnosis code of AA (441.1, 441.2, 441.3, 441.4, 441.5, 441.6, 441.7, and 441.9). The date when the coding of AA first appeared was defined as the index date. Those with previous aortic dissection (441.0, 441.00, 441.01, 441.02, and 441.03) were excluded. The accuracy of coded AA diagnoses in the NHIRD has been validated [20]. For each case, a pool of eligible controls with diagnosis of type 2 diabetes mellitus (DM) but without AA was created. The index data was the date of AA for the corresponding case. The same exclusion criteria were applied. From these eligible controls, one subject was selected randomly to match a case of AA according to duration of follow-up (cohort entry to index date), age (±5 years), sex, socioeconomic status, Charlson comorbidity index score (±3 score), adapted diabetes complications severity index score (±1 score), duration of diabetes mellitus (±3 months), and risk factor for AA including hypertension, myocardial infarction, cerebrovascular disease, chronic kidney disease, and peripheral artery disease.

### Exposure assessment

For the exposure of OADs, we identified variable classes of OADs, including metformin, sulfonylureas, TZD, alpha-glucosidase inhibitors, meglitinide, DPP-4 inhibitors (approved in Taiwan in 2009) at any time prior to the index date. We collected the following information for each OAD prescription, including dispensing date, drug type, quantity, and duration of drug supply. Besides, we also identified the concomitant drugs which potentially influence the risk of AA including alpha-blocker, angiotensin-converting-enzyme (ACE) inhibitor or angiotensin II receptor blockers (ARB), beta blocker, calcium channel blocker, diuretics, antiplatelet agent, warfarin, statin, steroid, antidepressants, nonsteroidal anti-inflammatory drugs (NSAID), and insulin. Moreover, we extracted the drug prescriptions retrospectively for the period extending to January 1997, and ensured that all individuals had available data for at least 3 years before study inclusion.

### Statistical analysis

The baseline demographics characteristics were compared between groups. For categorical variables, Chi square test was used for analysis. For continuous variables, independent *t* test or Mann–Whitney U test were used. Odds radios (ORs) were used to compare the exposure of OADs between cases and controls. For OAD users, cumulative dose was categorized into quintiles to explore the dose-response relationships. We conducted conditional logistic regression with adjustment for potential confounding factors, including prescriptions of alpha-blocker, ACE inhibitor or ARB, beta blocker, calcium channel blocker, diuretics, antiplatelet agent, warfarin, statin, steroid, antidepressants, NSAID, and insulin. Statistical significance was set at *p* < 0.05. For data linkage, processing, and sampling, we used the Microsoft SQL Server 2012 (Microsoft Corp., Redmond, Washington, USA). All analyses were performed using STATA statistical software (version 13.0; StataCorp., College Station, Texas, USA).

## Results

We had identified 4468 cases of AA and 4468 controls with DM diagnosis between 2000 and 2013. The baseline demographics were shown in Table 1. The mean age was 67.5 years and predominately male (66.5 %). While comparing other concomitantly prescribed medications, alpha-blockers, ACE inhibitors/ARBs, beta-blockers, calcium channel blockers, diuretics, antiplatelets, warfarins, statins, and anti-depressants were more prevalent among cases than among controls.

**Table 1:** Characteristics of the cases and controls

	Cases	Control	p value
Patients (no.)	4468	4468	
Age, mean (SD), years	67.5 (47.3)	67.5 (47.3)	>0.99
Male sex	2969 (66.5)	2969 (66.5)	>0.99
Monthly income			
Dependent	1614 (36.1)	1614 (36.1)	>0.99
0–19,100 NT dollars	1291 (28.9)	1291 (28.9)	
19,100–42,000 NT dollars	1454 (32.5)	1454 (32.5)	
> 42,000 NT dollars	109 (2.4)	109 (2.4)	
Urbanization^a^			
Level 1	1290 (28.9)	1290 (28.9)	>0.99
Level 2	3000 (67.1)	3000 (67.1)	
Level 3	158 (3.5)	158 (3.5)	
Level 4	20 (0.4)	20 (0.4)	
Charlson comorbidity index score^b^ (SD)	5.1 (2.6)	5.0 (2.5)	0.051
Adapted diabetes complications severity index score^c^ (SD)	2.7 (1.7)	2.6 (1.7)	0.057
Duration of diagnosis of diabetes mellitus, months (SD)	67.5 (47.3)	67.5 (47.3)	>0.99
Other comorbidity			
Hypertension	4049 (90.6)	4049 (90.6)	>0.99
Myocardial infarction	463 (10.4)	463 (10.4)	>0.99
Cerebrovascular disease	2261 (50.6)	2261 (50.6)	>0.99
Chronic kidney disease	1529 (34.2)	1529 (34.2)	>0.99
Peripheral artery disease	395 (8.8)	395 (8.8)	>0.99
Anti-hypertensive drug use			
Alpha blocker	1619 (36.2)	1501 (33.6)	0.009
ACE inhibitor or ARB	3538 (79.2)	3386 (75.8)	<0.001
Beta blocker	3571 (79.9)	3369 (75.4)	<0.001
Calcium channel blocker	3812 (85.3)	3596 (80.5)	<0.001
Diuretics	3345 (74.9)	3102 (69.4)	<0.001
Antiplatelet agent	3751 (84.0)	3630 (81.2)	0.001
Warfarin	271 (6.1)	193 (4.3)	<0.001
Statin	1976 (44.2)	1838 (41.1)	0.003
Antidepressants	1984 (44.4)	1826 (40.9)	0.001
Steroid	3870 (86.6)	3813 (85.3)	0.082
NSAID	4427 (99.1)	4426 (99.1)	0.912
Insulin	419 (9.4)	453 (10.1)	0.225

^a^Urbanization levels in Taiwan are divided into four strata according to the Taiwan National Health Research Institute publications. Level 1 designates the most urbanized areas, and level 4 designates the least urbanized areas

^b^Charlson comorbidity index (CCI) score is used to determine overall systemic health. With each increased level of CCI score, there are stepwise increases in the cumulative mortality

^c^Adapted diabetes complications severity index is a 13-point scale from 7 complication categories: retinopathy, nephropathy, neuropathy, cerebrovascular, cardiovascular, peripheral vascular disease, and metabolic, ranging from each complication. Each complication produced a numeric score ranging from 0 to 2 (*0* no abnormality, *1* some abnormality, *2* severe abnormality)

Table 2 presents the crude and adjusted ORs for the development of AA in association with OAD use compared with controls, after adjusting for all potential confounders in Table 1. Metformin use, sulfonylurea use, and TZD use were associated with lower risk of developing AA, adjusted OR 0.72 (95 % confidence interval [CI] 0.64–0.80), 0.82 (95 % CI 0.74–0.92), and 0.82 (95 % CI 0.69–0.98), respectively (Table 2). There was no association between developing of AA and alpha-glucosidase inhibitors (adjusted OR 0.95; 95 % CI 0.81–1.11) or DPP-4 inhibitors (adjusted OR 0.85; 95 % CI 0.68–1.07).

**Table 2:** Crude and adjusted rate ratios for the risk of aortic aneurysm with oral antidiabetic drugs

	No. (%)		Odds ratio (95 % CI)			
	Cases (n = 4468)	Control (n = 4468)	Crude	*p* value	Adjusted^a^	*p* value
No metformin use^b^	2882	2455	1 [Reference]		1 [Reference]	
Metformin use	1586	2013	0.64 (0.58–0.70)	<0.001	0.72 (0.64–0.80)	<0.001
No DPP-4 inhibitor use^b^	4305	4281	1 [Reference]		1 [Reference]	
DPP-4 use	163	187	0.85 (0.68–1.07)	0.168	1.07 (0.84–1.36)	0.582
No sulfonylurea use^b^	2779	2412	1 [Reference]		1 [Reference]	
Sulfonylurea use	1689	2056	0.68 (0.62–0.75)	<0.001	0.82 (0.74–0.92)	0.001
No alpha-glucosidase inhibitors use^b^	4077	3999	1 [Reference]		1 [Reference]	
Alpha-glucosidase inhibitors use	391	469	0.81 (0.70–0.93)	0.004	0.95 (0.81–1.11)	0.507
No thiazolidinedione use^b^	4171	4065	1 [Reference]		1 [Reference]	
Thiazolidinedione use	297	403	0.70 (0.59–0.82)	<0.001	0.82 (0.69–0.98)	0.003

^a^Adjusted for using alpha blocker, ACE inhibitor or ARB, beta blocker, calcium channel blocker, diuretics, antiplatelet agent, warfarin, statin, steroid, antidepressants, NSAID, and insulin

^b^Use of one prescription at any time prior to the index date

We further examine the dosage effect. While stratified by quantile according to the dose of OAD, the effect on AA was dose responsive for metformin (p for trend <0.001) and sulfonylurea (p for trend <0.001), but not for TZD (p for trend 0.431). The detailed results were shown in Table 3.

**Table 3:** Crude and adjusted rate ratios for the risk of aortic aneurysm with oral antidiabetic drugs

Dosage	No. (%)		Odds ratio (95 % CI)			
	Cases (n = 4468)	Control (n = 4468)	Crude	*p* value	Adjusted^a^	*p* value
No metformin use^b^	2882	2455	1 [Reference]		1 [Reference]	
Metformin use (days^c^)	1586	2013	0.64 (0.58–0.70)	<0.001	0.72 (0.64–0.80)	<0.001
Quantile 1 (11, 6–17)	364	363	0.85 (0.73–0.99)	0.042	0.91 (0.78–1.08)	0.291
Quantile 2 (56, 41–79)	326	400	0.66 (0.56–0.77)	<0.001	0.71 (0.60–0.85)	<0.001
Quantile 3 (181, 139–233)	303	404	0.60 (0.51–0.70)	<0.001	0.67 (0.56–0.81)	<0.001
Quantile 4 (459, 375–576)	316	404	0.60 (0.50–0.71)	<0.001	0.70 (0.58–0.86)	0.001
Quantile 5 (1127, 861–1666)	277	442	0.46 (0.38–0.55)	<0.001	0.57 (0.45–0.72)	<0.001
No DPP-4 inhibitor use^b^	4305	4281	1 [Reference]		1 [Reference]	
DPP-4 use (days^c^)	163	187	0.85 (0.68–1.07)	0.168	1.07 (0.84–1.36)	0.582
Quantile 1 (25, 14–30)	45	28	1.58 (0.98–2.55)	0.062	1.99 (1.20–3.29)	0.007
Quantile 2 (70, 56–84)	28	39	0.70 (0.43–1.15)	0.157	0.96 (0.57–1.62)	0.884
Quantile 3 (140, 112–168)	31	40	0.74 (0.46–1.20)	0.224	1.00 (0.61–1.65)	0.992
Quantile 4 (238, 210–273)	30	39	0.75 (0.46–1.22)	0.249	1.03 (0.62–1.72)	0.904
Quantile 5 (509, 413–672)	29	41	0.70 (0.43–1.13)	0.143	1.01 (0.60–1.68)	0.978
No sulfonylurea use^b^	2779	2412	1 [Reference]		1 [Reference]	
Sulfonylurea use (days^c^)	1689	2056	0.68 (0.62–0.75)	<0.001	0.82 (0.74–0.92)	0.001
Quantile 1 (20, 9–37)	402	359	0.96 (0.83–1.12)	0.624	1.04 (0.88–1.23)	0.630
Quantile 2 (120, 84–165)	336	401	0.70 (0.60–0.82)	<0.001	0.81 (0.69–0.97)	0.018
Quantile 3 (424, 313–544)	336	413	0.67 (0.57–0.79)	<0.001	0.79 (0.66–0.95)	0.010
Quantile 4 (1128, 881–1408)	312	437	0.55 (0.47–0.65)	<0.001	0.71 (0.59–0.87)	0.001
Quantile 5 (3164, 2329–4620)	303	446	0.51 (0.43–0.61)	<0.001	0.74 (0.59–0.94)	0.014
No alpha-glucosidase inhibitors use^b^	4077	3999	1 [Reference]		1 [Reference]	
Alpha-glucosidase inhibitors use (days^c^)	391	469	0.81 (0.70–0.93)	0.004	0.95 (0.81–1.11)	0.507
Quantile 1 (7, 5–10)	83	108	0.75 (0.56–1.00)	0.050	0.89 (0.66–1.21)	0.463
Quantile 2 (28, 19–35)	79	77	0.99 (0.72–1.36)	0.948	1.14 (0.82–1.59)	0.448
Quantile 3 (68, 56–85)	81	88	0.89 (0.66–1.22)	0.477	1.04 (0.76–1.44)	0.793
Quantile 4 (164, 134–210)	79	93	0.83 (0.61–1.12)	0.212	1.03 (0.75–1.42)	0.861
Quantile 5 (444, 344–630)	69	103	0.63 (0.45–0.87)	0.005	0.87 (0.61–1.23)	0.423
No thiazolidinedione use^b^	4171	4065	1 [Reference]		1 [Reference]	
Thiazolidinedione use (days^c^)	297	403	0.70 (0.59–0.82)	<0.001	0.82 (0.69–0.98)	0.003
Quantile 1 (19, 13–28)	71	69	0.97 (0.70–1.36)	0.873	1.12 (0.79–1.59)	0.518
Quantile 2 (65, 56–84)	54	86	0.58 (0.41–0.83)	0.003	0.77 (0.53–1.12)	0.167
Quantile 3 (206, 168–266)	54	87	0.59 (0.42–0.84)	0.003	0.77 (0.53–1.11)	0.164
Quantile 4 (532, 413–630)	65	74	0.83 (0.59–1.16)	0.281	1.14 (0.79–1.63)	0.485
Quantile 5 (1247, 977–1980)	53	87	0.57 (0.40–0.81)	0.002	0.80 (0.54–1.17)	0.241

^a^Adjusted for using alpha blocker, ACE inhibitor or ARB, beta blocker, calcium channel blocker, diuretics, antiplatelet agent, warfarin, statin, steroid, antidepressants, NSAID, and insulin

^b^Use of one prescription at any time prior to the index date

^c^Medians, interquartile range

## Discussion

In this retrospective, nested-case control study, we demonstrated the associations of diverse classes of OADs in development of AA. After adjustment, occurrence of AA remains lower in those receiving metformin, sulfonylurea, and TZD, but not DPP4 inhibitors and alpha-glucosidase inhibitors. Dose-response relationships are seen in metformin and sulfonylurea treated groups.

Thompson et al. conducted a prospective observational study for drug effects on AA growth in 2010 [21]. Of the 1296 patients followed for 3.4 years, exposure to hypoglycemic agents was associated with a slower AA growth rate (1.70 mm per year vs. 0.74 mm per year, estimated difference −0.95, 95 % CI −1.66 to −0.25). Since there were no differences between drug classes, he attributed the protective effect to DM, instead of OADs. An epidemiologic study assessing risk factors for AA growth also revealed a 0.11 cm decrease in AA diameter per year among diabetic patients [22]. Others disclosed significant lower incidence rate of AA and lower mortality in DM patients [23, 24]. However, the influence of OADs was not taken into consideration. In comparison, our study results show that patients receiving several classes of OADs, including metformin, sulfonylurea, and TZD have lower risks, but not in those treated with alpha-glucosidase inhibitor or DPP-4 inhibitor. Besides, there is a dose-response relationships and metformin and sulfonylurea treated patients. The differences between drug classes and dose-response relationships provide new evidence for the beneficial effects of OADs in AA.

Impacts of OADs on AA growth have been found in previous clinical and animal studies. Vasamsetti et al. demonstrated an attenuation of atheromatous plaque and AA formation in metformin treated ApoE(−/−) mice [11]. In the same experimental study, they found metformin induced AMPK activation. The subsequent inhibition of monocyte-to-macrophage differentiation and proinflammatory cytokine production may explain why metformin was able to protect the mice from aneurysm formation. More recently, Fujimura et al. reported that metformin is associated with a below-median enlargement rate in abdominal AA patients in a population-based study [25]. They also demonstrated that metformin dramatically inhibited the formation and progression of aneurysm in an experimental model, which was shown by preservation of smooth muscle and reduction of aortic mural macrophage, CD8 T cell, and neovascularity [25]. Similar effect was seen in TZDs. Pirianov et al. demonstrated rosiglitazone treated ApoE(−/−) mice with lower incidence of development and rupture of abdominal AA [26]. It was caused by inhibiting c-Jun N- terminal kinase phosphorylation and down-regulating toll-like receptor four expression at the lesion site, leading to a decrease of CD4 antigen and reduction in proinflammatory chemokines production.

There was no direct clinical or experimental study discussing the possible mechanism of sulfonylurea’s protective effect to date. Based on our current understanding of pharmacologic effect of sulfonylurea, we suggest that the SUR2 receptor, mainly expressed in the smooth muscle cell wall might play a role. An indirect evidence to this assumption was seen in a family of Cantu syndrome reported by Hiraki et al. [27]. They reported a family of Cantu syndrome, which is a genetic disorder characterized by *ABCC9* mutation, affecting both SUR2A and SUR2B. One of the family members was presented with AA.

DPP-4 inhibitors treated individuals were not beneficial with regard to AA occurrence in our cohort. This is discrepant to previous findings in animal studies. Bao et al. used alogliptin to treat their aneurysmal rats [13]. The alogliptin treated groups had a lower rate of aneurysm expansion, and fewer ROS, MMPs expression in aneurysm walls. Lu et al. also showed significantly fewer MMP-2 and MMP-9 production, associated with lower incidence of ApoE(−/−) mice [14]. In addition, the beneficial effect of DPP-4 inhibitors on major adverse cardiac events was shown in several studies [28, 29]. Since AA have been seen as a part of the spectrum of cardiovascular disease, we assumed DPP-4 inhibitor might also possess protective effects on AA. There are three possible causes to explain the discrepancy. First, the duration of exposure to DPP-4 inhibitor may not be long enough. The first DPP-4 inhibitor was approved in Taiwan in 2009. Our case collection ended in 2013. Thus, the patients had only been taken DPP-4 inhibitor for less than 4 years. A longer follow up period is probably needed. Second, the case number may be too small. In patients enrolled in our study, only 163 individuals were prescribed with DPP-4 inhibitors in the aneurysm group, and 187 in the control group. The relatively small sample size is not enough to achieve an adequate power.

The strength of the current study is the inclusion of large cases representing the nationwide diabetes populations from 2000 to 2013, which thus minimized referral bias. Still, this study has several limitations. First, unmeasured confounding is the primary limitation inherent in the use of administrative data. Some lifestyle information and cardiovascular risk indicators such as smoking status, alcohol consumption, obesity, dietary habits, exercise condition and lipid profiles were not available through the administrative dataset. However, due to low incidence of AA, it is difficult to conduct a randomize control study with sufficient power. Second, information on indices of diabetes control, such as glycosylated hemoglobin or fasting glucose level, was lacking. Nevertheless, the observed associations between the risk of AA and different OADs with similar glucose lowering effects and indications were not all in the same direction. If the influence of OADs on aneurysm formation was mainly from the effect of the glucose-lowering, the tendency of ORs in different OADs should tend toward coherence. Furthermore, the duration and severity (evaluated by adapted diabetes complications severity index score) of type 2 DM were matched between study groups (shown in Table 1). Thus, it is less likely that this unmeasured confounder biased the results. Third, relevant details regarding AA severity, such as AA diameter or annual rate of AA expansion, were not available in our administrative claims data set. AAs are often asymptomatic in early stages, and they are imperceptible for general population. For those, who may have small AA but without any symptom and without further diagnosis, would not have medical record in NHIRD. Hence, it may be underestimated the true prevalence and incidence of AA in Taiwan. Fourth, on account of the present retrospective and observational study design, we were unable to determine the direction of causality. Finally, comparing with epidemiological studies in Western countries, our study showed a relatively low prevalence among Taiwanese population, which was consistent with previous studies from Asian countries (0.3–0.5 %) [30]. However, the prevalence and incident rate in Taiwan NHI database were even lower than reported rates among other Asian countries [30]. The prevalence calculated from diagnosis-specific claim data might be lower than those epidemiological data from the reports of screening programs. Therefore, the results from the present study are not necessarily applicable on Caucasian populations given the lower prevalence of AA in Asian populations.

## Conclusions

Oral antidiabetic agents, including metformin, sulfonylurea, and TZD showed protective effects on abdominal AA development, but not DPP-4 inhibitors or alpha-glucosidase inhibitor. The protective effects of OADs are further confirmed by the dose responsive relations in metformin and sulfonylurea groups. In the future, well-conducted prospective studies are necessary to give stronger evidence of the OADs protective effects on AA.

## Abbreviations

OAD: oral antidiabetic drugs
AA: aortic aneurysm
TZD: thiazolidinedione
DPP-4: dipeptidyl peptidase-4
OR: odds ratio
CI: confidence interval
MMP: matrix metalloprotein
ROS: reactive oxygen species
ApoE: apolipoprotein-E
NHI: National Health Insurance
NHIRD: National Health Insurance Research Database
DM: diabetes mellitus
ACE: angiotensin-converting-enzyme
ARB: angiotensin II receptor blockers
NSAID: nonsteroidal anti-inflammatory drugs
